# WEE1 Inhibition Enhances Anti-Apoptotic Dependency as a Result of Premature Mitotic Entry and DNA Damage

**DOI:** 10.3390/cancers11111743

**Published:** 2019-11-07

**Authors:** Mathilde Rikje Willemijn de Jong, Myra Langendonk, Bart Reitsma, Pien Herbers, Marcel Nijland, Gerwin Huls, Anke van den Berg, Emanuele Ammatuna, Lydia Visser, Tom van Meerten

**Affiliations:** 1 Department of Hematology, University Medical Center Groningen, University of Groningen, 9713 GZ Groningen, the Netherlandsm.langendonk@umcg.nl (M.L.); a.a.reitsma@umcg.nl (B.R.); pienherbers@live.nl (P.H.); m.nijland@umcg.nl (M.N.); g.huls@umcg.nl (G.H.); e.ammatuna@umcg.nl (E.A.); 2 Department of Pathology and Medical Biology, University Medical Center Groningen, University of Groningen, 9713 GZ Groningen, the Netherlands; a.van.den.berg01@umcg.nl (A.v.d.B.); l.visser@umcg.nl (L.V.)

**Keywords:** diffuse large B-cell lymphoma, BH3 profiling, WEE1, AZD1775, venetoclax, navitoclax

## Abstract

Genomically unstable cancers are dependent on specific cell cycle checkpoints to maintain viability and prevent apoptosis. The cell cycle checkpoint protein WEE1 is highly expressed in genomically unstable cancers, including diffuse large B-cell lymphoma (DLBCL). Although WEE1 inhibition effectively induces apoptosis in cancer cells, the effect of WEE1 inhibition on anti-apoptotic dependency is not well understood. We show that inhibition of WEE1 by AZD1775 induces DNA damage and pre-mitotic entry in DLBCL, thereby enhancing dependency on BCL-2 and/or MCL-1. Combining AZD1775 with anti-apoptotic inhibitors such as venetoclax (BCL-2i) or S63845 (MCL-1i) enhanced sensitivity in a cell-specific manner. In addition, we demonstrate that both G2/M cell cycle arrest and DNA damage induction put a similar stress on DLBCL cells, thereby enhancing anti-apoptotic dependency. Therefore, genotoxic or cell cycle disrupting agents combined with specific anti-apoptotic inhibitors may be very effective in genomic unstable cancers such as DLBCL and therefore warrants further clinical evaluation.

## 1. Introduction

Diffuse large B-cell lymphoma (DLBCL) is a genomically unstable cancer with multiple low-frequency mutations, somatic copy number alterations, and chromosomal translocations [1]. To survive in such a genetically hazardous setting, DLBCL cells rely on cell cycle checkpoints, DNA repair, and anti-apoptotic proteins [2,3]. We have recently demonstrated that the cell cycle regulator WEE1 is highly expressed in DLBCL and is a relevant target for therapy [4]. WEE1 acts through phosphorylation of cyclin-dependent kinase 1 (CDK1/CDC2), which blocks the cell cycle at G2/M to allow time for DNA damage surveillance [5]. Under normal circumstances, factors such as cell cycle disruption and DNA damage result in induction of (intrinsic) apoptosis [6,7,8,9,10]. Indeed, blocking of WEE1 activity with AZD1775 results in apoptosis in solid cancers [11], lymphoma [4], and leukaemia [12,13]. In order to survive intrinsic apoptosis, DLBCL and many other cancers induce upregulation of various anti-apoptotic proteins, leading to apoptosis resistance, resulting in a cellular reliance on anti-apoptotic proteins commonly termed ‘anti-apoptotic dependency’ [14,15]. Since AZD1775 is very effectively able to induce intrinsic apoptosis, we aimed to investigate whether AZD1775 could alter the anti-apoptotic dependency and enhance the sensitivity of DLBCL cells to anti-apoptotic inhibitors (a.k.a., BH3 mimetic drugs).

The intrinsic apoptotic pathway is regulated by a complex interaction of pro- and anti-apoptotic proteins at the surface of mitochondria. Cells undergo apoptosis when this protein complex is balanced towards pro-apoptotic proteins (e.g., BIM (pro-apoptotic effector), BAD, NOXA, PUMA, BMF, HRK (pro-apoptotic sensitizers)). On the other hand, when apoptotic dependency shifts towards anti-apoptotic proteins (e.g., BCL-2, BCL-XL, BCL-W, and MCL-1) cells can avoid apoptosis [16]. Many studies in both cell lines and primary patients’ samples have demonstrated the (clinical) potential of BH3 profiling, a technique that can identify a selective dependency on individual pro- or anti-apoptotic proteins in this complex network [17]. With this method, cells are exposed to BH3 peptides which enter the mitochondria and interact with anti-apoptotic proteins, resulting in a mitochondrial outer membrane permeabilization (MOMP) which can be measured using the JC-1 dye or as cytochrome-c release. Dynamic BH3 profiling (dBH3) is an ex vivo, clinically applicable variant of BH3 profiling which measures early changes in the apoptotic signalling cascade at the mitochondrial level, whether induced in cancer cells by treatment with classical chemotherapy or by specific anti-cancer agents [18,19,20].

In the present study we showed that WEE1 inhibitor AZD1775 induces cell death through apoptosis. In addition, we showed that AZD1775 significantly enhanced the dependency on anti-apoptotic proteins and enhanced sensitivity to BH3 mimetic drugs in DLBCL cells in a cell-specific manner. Similar changes in the anti-apoptotic dependency were induced by DNA damage and cell cycle arrest independently. Based on these results, we predict that AZD1775 and other similar drugs are very suitable to combine with BH3 mimetic drugs for the treatment of cancer.

## 2. Results

### 2.1. AZD1775 Induces Apoptosis and Enhances Anti-Apoptotic Dependency in DLBCL

**Figure 1:**
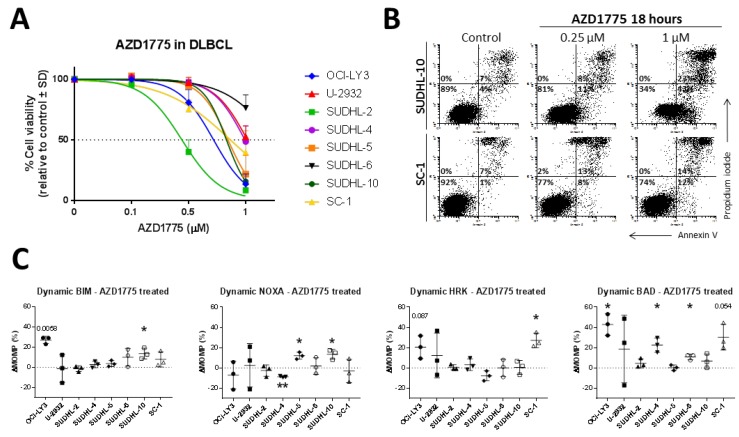
Apoptosis induced by AZD1775 in diffuse large B-cell lymphoma (DLBCL). (**A**) Cell viability analysis was measured with propidium iodide flow cytometry in DLBCL cell lines treated with AZD1775 for 72 h. Data were plotted as the mean ± SD (*n* = 3). (**B**) Representative flow cytometry of apoptosis (Annexin V/Propidium iodide) induced by treatment of AZD1775 for 18 h in representative cell lines SUDHL-10 and SC-1. (**C**) Mitochondrial response of DLBCL cell lines treated with AZD1775 for 18 h, plotted as the delta mitochondrial outer membrane permeabilization (ΔMOMP%). ΔMOMP% was calculated by subtracting the percentage treated MOMP from percentage untreated MOMP. Cell line SUDHL-10 was treated with 0.25 µM AZD1775, cell lines OCI-LY3, U-2932, SUDHL-2, and SUDHL-5 were treated with 0.5 µM AZD1775 and cell lines SUDHL-4, SUDHL-6, and SC-1 were treated with 1 µM AZD1775. Data were plotted as the mean ± SD (*n* = 3). Statistical analysis was performed using a one-sample *t*-test as compared to untreated control cells (* *p* ≤ 0.05).

DLBCL is a genomic unstable and heterogeneous cancer that shows a variety of molecular phenotypes. We previously demonstrated high expression of WEE1 in DLBCL cells compared to normal B-cells [4] and found this difference is significantly higher in DLBCL compared to other cancers (Figure S1). These data suggest that genomically unstable tumours such as DLBCL might be particularly sensitive to WEE1 inhibition. Thereto, we selected a panel of DLBCL cell lines with different genetic aberrations (Table S1). Treatment of DLBCL cell lines with WEE1 inhibitor AZD71775 induced a rapid decline in cell viability, with IC50 values ranging from 0.4 to 1.8 µM (Figure 1A and Table S1).

Cell death was induced by apoptosis, as measured by flow cytometry for annexin V/PI staining (Figure 1B). Apoptosis induced by AZD1775 could be observed in both a time and dose-dependent manner and could be rescued by pan-caspase inhibitor QVD (Figure S2). Next, we used dynamic BH3 profiling (Figure 1C and Table S2), which measures the changes in anti-apoptotic dependency upon treatment with AZD1775. As a result of AZD1775 treatment, cell lines OCI-LY3, SUHDL-6, SUDHL-10, and SC-1 had increased mitochondrial response to the pro-apoptotic peptide BIM at 27% (*p* = 0.0058), 10% (ns), 14% (*p* = 0.0480), and 8% (ns), respectively, indicating cells were more primed to undergo apoptosis. BH3 profiling with a mean ΔMOMP ≥ 20% was classified as biologically relevant, even if they were not statically significant, as they often lead to significant enhanced sensitivity to BH3 mimetic drugs, indicating biological relevance. In addition, the changes induced by AZD1775 treatment could be induced in a dose-dependent manner (Figure S3A) and were significantly correlated to the percentage of apoptotic cells (Figure S3B,C). To investigate whether AZD1775-treated cells try to resist apoptosis, we next studied the dynamics of anti-apoptotic proteins MCL-1, BCL-XL, and BXL-2 in response to WEE1 inhibition as measured by mitochondrial response for NOXA, HRK, and BAD, respectively (Figure 1C). A significantly increased mitochondrial response to NOXA was observed in SUDHL-5 (12%, *p* = 0.0256) and in SUDHL-10 (13%, *p* = 0.0393), indicating increased dependency on MCL-1 upon AZD1775 treatment. For cell lines OCI-LY3 and SC-1, a significantly increased response was observed for HRK (21%; *p* = 0.0866 and 27%; *p* = 0.0223, respectively), demonstrating WEE1 inhibition increased dependency on BCL-XL. Most cell lines showed an increased mitochondrial response to BAD upon AZD1775 treatment, which reached 43% in OCI-LY3 (*p* = 0.0195), 20% in U-2932 (ns), 23% in SUDHL-4 (*p* = 0.0317), 11% in SUDHL-6 (*p* = 0.0223), and 30% in SC-1 (*p* = 0.0540). These results suggest that AZD1775 treatment leads to an increased dependency on BCL-2/BCL-XL/BCL-W. Only in cell line SUDHL-2, AZD1775 treatment did not induce changes in the anti-apoptotic dependency (Figure 1C), despite being relatively sensitive to AZD1775 treatment (Figure 1A). These results suggest that perhaps other apoptosis pathways, such as the extrinsic pathway, might be involved in the induction of apoptosis in SUDHL-2. Dynamic changes induced by AZD1775 in the different DLBCL cell lines showed no significant differences dependent on the GCB or ABC-subtype of DLBCL cell lines nor the TP53 status (Table S1). In conclusion, AZD1775 induces cell death through apoptosis, which enhanced the dependency on anti-apoptotic proteins.

### 2.2. DNA Damage and Premature Mitotic Entry Induced by AZD1775 Enhance Anti-Apoptotic Dependency

**Figure 2:**
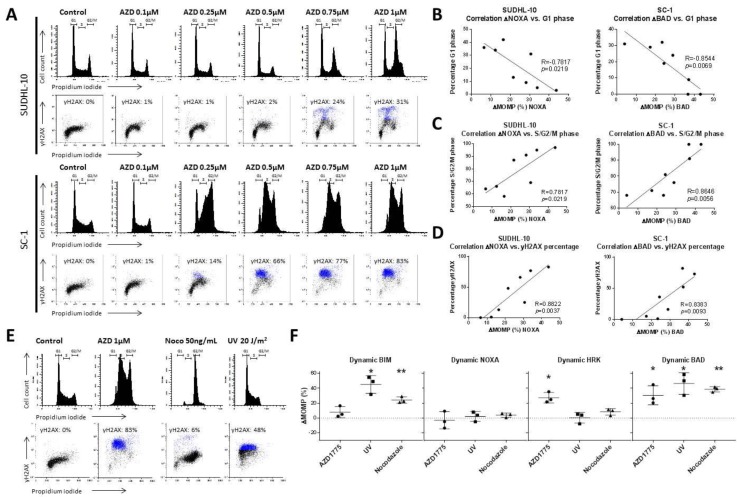
Cellular effect of AZD1775 treatment in DLBCL. (**A**) Representative examples of flow cytometry cell cycle and γH2AX analysis of AZD1775 in SUDHL-10 and SC-1 treated for 18 h. (**B**) Correlation of the mitochondrial response (ΔMOMP%) for 10 µM NOXA in SUDHL-10 or 0.1 µM BAD in SC-1 versus the percentage of G1 phase cells treated with AZD1775. (**C**) Correlation of the mitochondrial response (ΔMOMP%) for 10 µM NOXA in SUDHL-10 or 0.1 µM BAD in SC-1 versus the percentage of S/G2/M phase cells treated with AZD1775. (**D**) Correlation of the mitochondrial response (ΔMOMP%) of 10 µM NOXA in SUDHL-10 or 0.1 µM BAD in SC-1 versus the percentage of γH2AX cells treated with AZD1775. Cells were treated with 0, 0.1, 0.2, 0.3, 0.4, 0.5, 0.75, or 1.0 µM AZD1775 for 18 h. (**E**) Representative examples of cell cycle flow cytometry and γH2AX analysis in SC-1 treated with 1 µM AZD1775, 50 ng/mL nocodazole, and 20 J/m^2^ UV radiation. (**F**) Dynamic BH3 profile for 0.3 µM BIM, 10 µM NOXA, 10 µM HRK, and 0.1 µM BAD of SC-1 cells treated with AZD1775, nocodazole, and (ultra violet) UV radiation. Data were plotted as the mean ± SD (*n* = 3). Statistical analysis was performed using a one-sample *t*-test as compared to untreated control cells (* *p* ≤ 0.05).

WEE1 inhibition by AZD1775 induces both premature mitotic entry [21,22] and DNA damage [23] in various types of cancer cells. To confirm that AZD1775 has a similar effect on DLBCL cells, we analysed cell cycle distribution and γH2AX expression as a marker for DNA damage in the representative MCL-1 dependent cell line SUDHL-10 and the BCL-2 dependent cell line SC-1 (Figure 2A). AZD1775 treatment resulted in a dose-dependent increase in the percentage of G2/M cells, indicating premature mitotic entry and/or prolonged mitotic arrest and a dose-dependent increase of DNA damage (γH2AX positive cells) in both SUDHL-10 and SC-1 (Figure 2A).

In addition, dynamic BH3 profiling showed a dose-dependent correlation between mitochondrial response to BAD in SC-1 cells treated with AZD1775 and the decrease in the percentage of G1 phase cells (R = −0.8544, *p* = 0.0069) (Figure 2B), an increase in the percentage of S/G2/M phase cells (*R* = 0.8646, *p* = 0.0056) (Figure 2C), and an increase in γH2AX levels (*R* = 0.8383, *p* = 0.0093) (Figure 2D). Similar correlation for response to NOXA and G-1 phase cells (*R* = −0.7817, *p* = 0.0219), percentage of S/G2/M phase cells (*R* = 0.9036, *p* = 0.0021), and γH2AX (*R* = 0.8822, *p* = 0.0037) were observed in SUDHL-10. These results indicate a potential relation between G2/M cell cycle arrest and increased DNA damage that could affect the anti-apoptotic dependency in DLBCL cells.

Next, we investigated if cell cycle distribution and/or DNA damage could induce changes in anti-apoptotic dependency, by exploring the ability of nocodazole to induce cell cycle arrest and of ultraviolet radiation (UV) to induce γH2AX (Figure 2E,F and Figure S4) in SC-1 cells. Nocodazole treatment induced an increase in the percentage of cells in the G2/M phase and a moderate (6%) increase in γH2AX-positive cells. UV exposure had a limited effect on the cell cycle distribution, but induced γH2AX in about 60% of the cells (Figure 2E and Figure S4). Dynamic BH3 profiling revealed that both nocodazole and UV exposure induced a significant increase in the mitochondrial response to BIM, of 24% (*p* = 0.0249) and 45% (*p* = 0.0224), respectively (Figure 2F). Similarly, response levels to BAD significantly increased, at 39% (*p* = 0.0027) and 46% (*p* = 0.0307), respectively.

**Figure 3:**
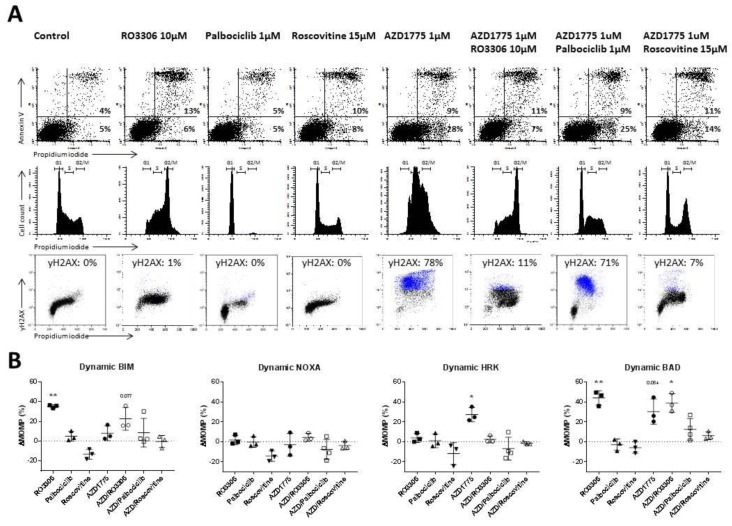
Rescue of AZD1775-induced cellular effects by CDK inhibitors RO3306, palbociclib and roscovitine. (**A**) Representative examples of apoptotic cells, cell cycle, and γH2AX flow cytometry analysis of SC-1 treated with 10 µM RO3306, 1 µM palbociclib, 15 µM roscovitine, and 1 µM AZD1775 after 18 h of incubation. (**B**) Dynamic BH3 profile for 0.3 µM BIM, 10 µM NOXA, 10 µM HRK, and 0.1 µM BAD of SC-1 cells treated with RO3306, palbociclib, roscovitine, and AZD1775 after 18 h of incubation. Delta mitochondrial outer membrane permeabilization (ΔMOMP%) was calculated by subtracting the percentage treated MOMP from percentage untreated MOMP. Data were plotted as the mean ± SD (*n* = 3). Statistical analysis was performed using a one-sample *t*-test as compared to untreated control cells (* *p* ≤ 0.05).

To further test the hypothesis that WEE1 inhibition induces changes in anti-apoptotic dependency as a result of cell cycle changes and DNA damage, we tested the combination of AZD1775 together with the CDK1 inhibitor RO3306, CDK4/6 inhibitor palbociclib, and CDK1/2 inhibitor roscovitine (Figure 3 and Figure S4), which has previously been shown to rescue AZD1775-induced DNA damage [24,25].

Treatment of SC-1 cells with the (single agent) roscovitine had little effects on either apoptosis levels, the cell cycle, or levels of DNA damage (Figure 3A,B). However, when combined with AZD1775, roscovitine significantly reduced DNA damage levels (from 75% to 7%, *p* = 0.0024) and largely prevented alterations in cell cycle distribution (Figure 3A,B). Treatment with roscovitine alone or in combination with AZD1775 did not lead to an altered dynamic BH3 profile, demonstrating that a full rescue of both cell cycle distribution and DNA damage prevents anti-apoptotic changes induced by AZD1775 (Figure 3B). In addition, we tested CDK1 inhibitor RO3306 (Figure 3A,B and Figure S5), which rescues only AZD1775-induced DNA damage, and CDK4/6 inhibitor palbociclib (Figure 3A,B and Figures S5 and S6), which rescues only AZD1775-induced cell cycle arrest. Neither partial rescue of DNA damage (Figure S4) nor cell cycle distribution (Figure 3A) were sufficient to prevent anti-apoptotic changes induced by AZD1775. Based on these results, we conclude that both DNA damage and cell cycle disruption induced independently changes in the mitochondria and enhanced anti-apoptotic dependency.

### 2.3. AZD1775 Increases Sensitivity to BCL-2 and MCL-1 Inhibitors in DLBCL

**Figure 4:**
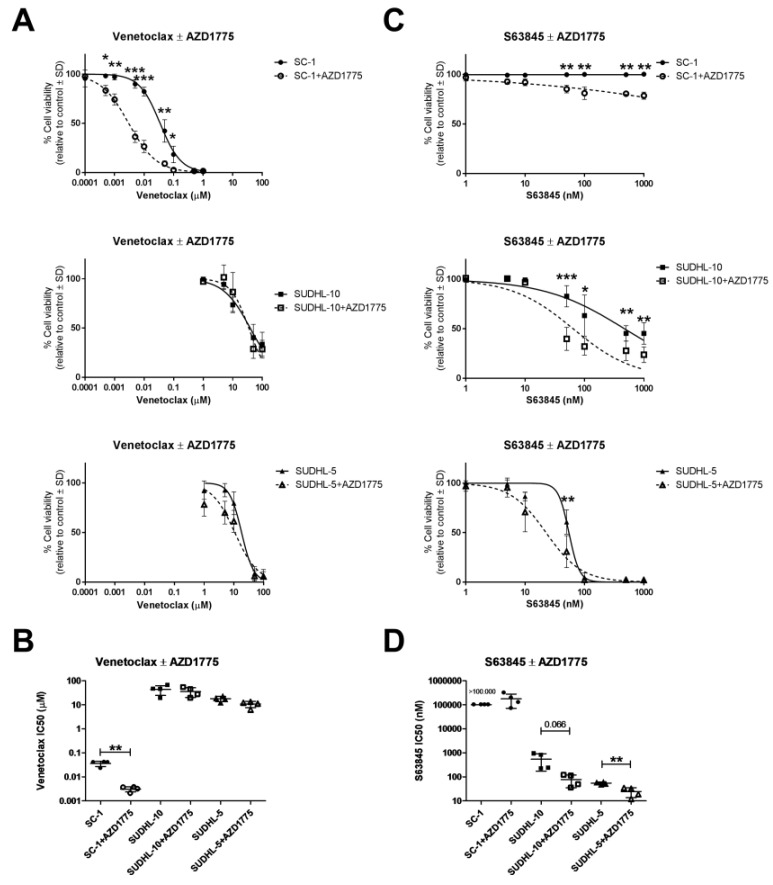
Combination of AZD1775 and BH3 mimetic drugs in DLBCL cell lines. (**A**) Cell viability flow cytometry data for the cell lines SC-1, SUDHL-5, and SUDHL-10 pre-treated with 1 µM AZD1775 and incubated with increasing concentrations of venetoclax. Data were normalized to the control and plotted as the mean ± SD (*n* = 4). (**B**) Venetoclax IC50 values as calculated from cell viability curves. Data were plotted as the mean ± SD (*n* = 4). (**C**) Cell viability flow cytometry data for SC-1, SUDHL-5, and SUDHL-10 pre-treated with 1 µM AZD1775 and incubated with increasing concentrations of S63845. Data were normalized to the control and plotted as the mean ± SD (*n* = 4). (**D**) S63845 IC50 values as calculated from cell viability curves. Data plotted as the mean ± SD (*n* = 4). Statistical analysis was performed using a two-tailed paired *t*-test (* *p* ≤ 0.05) (** *p* ≤ 0.01).

Now that we have established that DNA damage and cell cycle disruption by AZD1775 (Figure 2) is sufficient to induce intrinsic apoptosis (Figures S1–S3) and enhance dependency on anti-apoptotic proteins to prevent apoptosis, we next studied the combination of AZD1775 with anti-apoptotic inhibitors. We used SC-1 as a cell line with enhanced dependency on BCL-2/BCL-XL/BCL-W, and SUDHL-10 and SUDHL-5 as cell lines with enhanced dependency on MCL-1 as predicted by BH3 profiling (Figure 1C). Combined treatment of AZD1775 and venetoclax caused a 10-fold decrease in cell viability in SC-1 (Figure 4A).

A significant decline in viability from 82% to 26% (*p* < 0.0001) was observed when 0.01 µM venetoclax was combined with 1 µM AZD1775, resulting in an IC50 decrease from 0.04 µM in AZD1775-untreated cells to 0.003 µM in AZD1775-treated cells for venetoclax (*p* = 0.0039, Figure 4B). In the MCL-1 dependent cell lines SUDHL-10 and SUDHL-5, as expected, co-treatment with AZD1775 did not enhance sensitivity to venetoclax. In these cell lines, AZD1775 induced enhanced dependency on MCL-1, suggesting cells would become more sensitive to MCL-1 inhibitor S63845. Combined treatment of 1 µM AZD1775 and S63845 indeed caused a dose-dependent decrease in cell viability in the cell line SUDHL-5 (from 61% to 31% (*p* = 0.0024) at 50 nM S63845) and in cell line and SUDHL-10 (from 83% to 40% (*p* = 0.0003) at 50 nM S63845) (Figure 4C). These changes resulted in a 7-fold decrease in IC50 values for S63845 in SUDHL-10 (from 544 to 77 nM, *p* = 0.0658) and a 2-fold decrease in IC50 values for S63845 in SUDHL-5 (from 61 to 35 nM, *p* = 0.0028) (Figure 4D). No effect on cell viability was observed when AZD1775 was combined with S63845 in SC-1 (Figure 1C). These results confirm that the changes observed in dynamic BH3 profiling upon AZD1775 treatment enhance dependency on anti-apoptotic proteins, a dependency that can be effectively exploited through targeting with the appropriate BH3 mimetic.

### 2.4. AZD1775 Alters the Anti-Apoptotic Dependency of Patient-Derived DLBCL Cells

**Figure 5:**
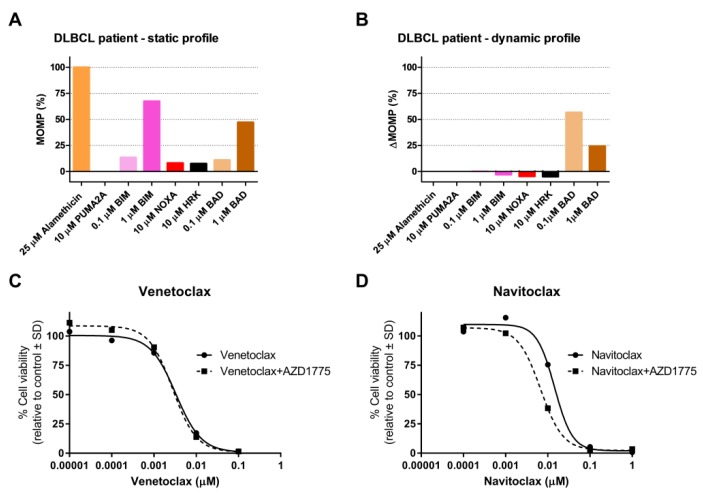
Dynamic BH3 profile and validation experiments in a DLBCL patient treated with AZD1775. (**A**) Static BH3 profile and (**B**) dynamic BH3 profile of DLBCL patient cells treated with 0.5 µM AZD1775 for 18 h. Alamethicin and PUMA2A were used as positive and negative controls, respectively. Delta mitochondrial outer membrane permeabilization (ΔMOMP%) was calculated by subtracting the percentage treated MOMP from percentage untreated MOMP. (**C**) Cell viability flow cytometry data for DLBCL patient cells pre-treated with AZD1775 for 18 h and incubated with increasing concentrations of venetoclax and (**D**) navitoclax for 24 h. Data were normalized to the control.

Finally, we studied anti-apoptotic dependency and the dynamic BH3 profile following AZD1175 treatment of lymphoma cells from a DLBCL patient (Figure S7). Efficient apoptotic priming was observed (Figure 5A), and dynamic BH3 profiling (Figure 5B) showed an increase in the mitochondrial response to BAD (56% at 0.1 µM BAD) but not to HRK after AZD1775 treatment, suggesting an increased sensitivity to BCL-2 or BCL-W inhibitors, but not to BCL-XL inhibitors.

These findings were validated by ex vivo treatment of lymphoma cells with AZD1775 at increasing concentrations of venetoclax (BCL-2i) or navitoclax (BCl-2i, BCl-Xli, and BCL-Wi). Venetoclax did not alter cell viability (Figure 5C), but navitoclax induced a 2-fold decrease in cell viability (Figure 5D), together with a decrease in IC50 from 0.015 µM for navitoclax alone to 0.0067 µM for navitoclax combined with AZD1775, indicating a dependency on BCL-W. We were unable to study the effect of BCL-W inhibition, as specific BCL-W inhibitors are not currently available. Taken together, these data emphasize the potential of AZD1775 combined with BH3 mimetics in the treatment of DLBCL patients and underscore the clinical utility of BH3 profiling.

## 3. Discussion

**Figure 6:**
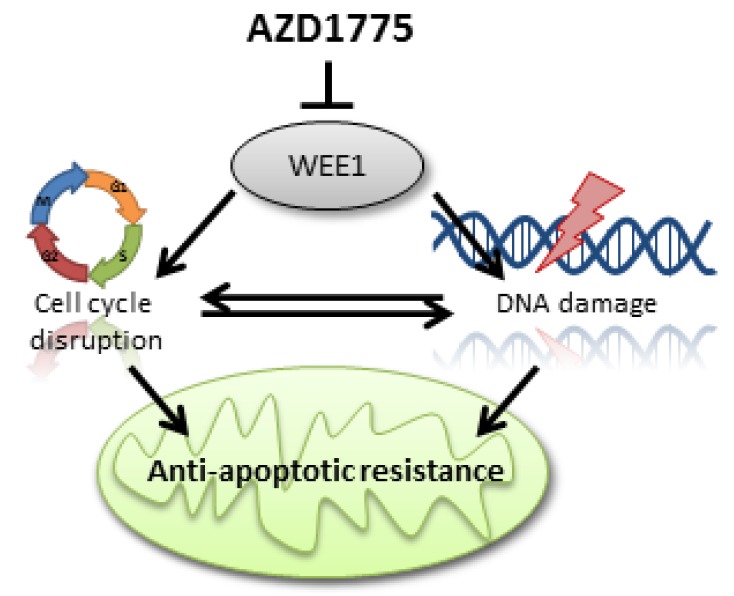
Proposed mechanism for the relation between inhibition of cell cycle regulator WEE1 and the resistance of apoptosis. As a result of WEE1 inhibition cells are able to prematurely enter mitosis, without proper repair of genomic aberrations or mutations. This elimination of the checkpoint at the transition from the G2-phase into the M-phase leads to continuous cell cycling and results in high levels of DNA damage that activate apoptosis pathways. To prevent apoptosis, cancerous cells become increasingly dependent on anti-apoptotic proteins such as BCL-2, MCL-1, BCL-XL, and BCL-W, which ensure survival under high levels of genomic stress.

In genomically unstable cancers such as DLBCL, WEE1 is highly expressed and a relevant target for therapy. Since the WEE1 inhibitor AZD1775 is indeed effective in inducing apoptosis we investigated if it could alter anti-apoptotic dependency and increase sensitivity to BH3 mimetic drugs in DLBCL cell lines and patient material. Our findings demonstrate that (1) AZD1775 induces cell death through apoptosis; (2) AZD1775-mediated inhibition of WEE1 alters the anti-apoptotic dependency in DLBCL; (3) combination of AZD1775 with cell-specific anti-apoptotic inhibitors (such as venetoclax) leads to enhanced potency; (4) both DNA damage and G2/M arrest induced by WEE1 inhibition independently enhances dependency on anti-apoptotic proteins. Based on these results, we propose a model in which cell cycle disruption (such as premature mitotic entry or G2/M arrest) and DNA damage can induce changes in the mitochondrial response, resulting in an altered dependency on anti-apoptotic proteins in DLBCL cells (Figure 6).

In normal B-cell development, dependency on anti-apoptotic proteins changes during B-cell maturation. Naïve B-cells are dependent on BCL-2, GC B-cells shift to dependency on MCL-1, memory B-cells are once more dependent on BCL-2, while plasma cells depend on BCL-XL [26]. The t(14;18) IGH-BCL-2 translocation, found in ~20% of DLBCL patients, is a hallmark of follicular lymphoma and is more commonly found in the germinal centre derived-DLBCL subtype [14,27]. BCL-W has a potentially important role in B-cell survival, as overexpression of the BCL-W gene is associated with a worse prognosis in specific DLBCL cases [28]. Using baseline BH3 profiles and sensitivity to BH3 mimetic drugs, we showed that DLBCL cell lines are dependent on multiple anti-apoptotic proteins. These associations showed no direct relationship to the cell of origin, thus highlighting the heterogeneity of DLBCL.

To date, the primary application of dynamic BH3 profiling has been measurement of responses to chemotherapy [17]. Here, we were able to accurately predict changes in anti-apoptotic dependency profiles as a result of WEE1 inhibition by AZD1775. These dependencies changed both quantitatively and qualitatively. For example, dynamic BH3 profiling highlighted interesting shifts in dependency, such as the shift to dependency on BCL-XL after AZD1775 treatment of the previously BCL-XL independent SC1 cell line. This shows that tumour cells can shift their dependency from one anti-apoptotic protein to another. Unfortunately, current multiple anti-apoptotic protein inhibitors, including those targeting BCL-XL such as navitoclax, are poorly tolerated and have significant side effects [29]. Inhibition of BCL-XL also shortens platelet lifespan [30], hampering use of these agents. Nevertheless, as tumour cells appear able to use and adapt their dependency to multiple anti-apoptotic proteins, there is clearly a need for new, tolerated multi-target inhibitors in order to prevent resistance and relapse in DLBCL patients. Our results demonstrate the broad range of effects of AZD1775 on cellular states, including the ability to influence apoptotic pathways, clearly highlighting potential therapeutic targets. It can be argued that analyses of protein levels by western blot or immunohistochemistry, perhaps including RNA expression levels, are suboptimal approaches when determining the anti-apoptotic dependency of tumour cells. By contrast, static BH3 profiling of cell lines or primary patient samples has proven itself as a fast and reliable tool to establish the functionality of and dependency on anti-apoptotic proteins.

Treatment of solid cancers [11] and leukaemia [12,13] with inhibitors of the cell cycle regulator WEE1 has proven successful in early clinical trials, as WEE1 inhibitors result in cell cycle disruption [21,22], induction of DNA damage [23,31] and eventually, induction of apoptosis. Our data highlighted a novel effect of WEE1 inhibition, an altered dependency on anti-apoptotic proteins. Rescue experiments preventing either DNA damage and/or G2/M cell cycle arrest after WEE1 inhibition showed that both events are able to induce changes in anti-apoptotic dependency, either individually or in combination. Mitochondrial fission at mitosis is known to be tightly regulated by the CDK1-cyclinB complex, which phosphorylates the GTPase Drp1 and thus induces mitochondrial fragmentation to promote mitochondrial fission [32,33]. Loss of Drp1 results in dysfunctional mitochondrial fission, persistent mitochondrial hyperfusion, delayed G2/M cell cycle progression, replication stress, DNA damage, ATM activation, and genomic instability [34]. Since WEE1 is the main regulator of the CDK1-cyclinB complex, loss of WEE1 will induce premature mitochondrial fragmentation, destabilizing the connection between cell cycle division and mitochondrial homeostasis. Similarly, activation of the DNA damage response protein ATM in response to double-stranded DNA breaks results in phosphorylation of the BH3 activator protein BID, initiating DNA damage-induced apoptosis [35]. Here, we show a novel direct link between the cell cycle, DNA damage and mitochondrial apoptosis, in which either premature mitotic entry or DNA damage induced by WEE1 inhibition alters anti-apoptotic dependency. Although this mechanism was not tested in other cancer types, based on the fundamental role of WEE1 in cell cycle regulation we expect a similar association and response in other cell types. With the current development of BH3 mimetics, WEE1 inhibition would be an excellent candidate for dose reduction combination therapy in treatment-naïve patients, or could be applied as a BH3 mimetic sensitizer in WEE1 inhibitor-resistant tumours.

In conclusion, we demonstrated for the first time that WEE1 inhibition in DLBCL can lead to further sensitization to anti-apoptotic inhibitors, revealing a novel mechanism and as yet unexplored application for WEE1 inhibition. Using cell cycle and DNA damage rescue experiments, we unraveled the mechanism underlying changes in anti-apoptotic dependency, highlighting mechanisms that may have applications in the treatment of other cancers. These findings suggest important applications for WEE1 as a novel therapeutic treatment approach, but also suggest broader possibilities for genotoxic drugs, cell cycle deregulators, or DNA damage response inhibitors in combination with BH3 mimetic drugs. Finally, we showed that combining WEE1 inhibition with dynamic BH3 profiling represents an educated approach to guided therapy, and may lead to novel strategies in optimalized/personalized treatment selection in DLBCL patients.

## 4. Materials and Methods

### 4.1. Cell Lines and Culture Conditions

The DLBCL cell lines U-2932 (ABC), SUDHL-2 (ABC), SUDHL-4 (GCB), and SC-1 (GCB) were cultured in suspension in Roswell Park Memorial Institute medium 1640 (RPMI 1640; Lonza BioWhittaker, Walkersville, MD, USA) with 10% fetal bovine serum (FBS; HyClone Thermo Scientific, Waltham, MA, USA), 1% Penicillin-Streptomycin (PS; Lonza BioWhittaker) and 1% glutamine (Lonza BioWhittaker). The DLBCL cell lines OCILY3 (ABC), SUDHL-5 (GCB), SUDHL-6 (GCB), and SUDHL-10 (GCB) were cultured in suspension in RPMI 1640 with 20% FBS, 1% PS and 1% glutamine. All cell lines were cultured at 37 °C with 5% CO2 in a humidified atmosphere. The identity of our cell lines was checked on a regular basis. Cell line characteristics are given in Table S1.

### 4.2. Patient Material and METC Statement

Patient material was acquired in accordance with international regulations and professional guidelines (the Declaration of Helsinki and the International Conference on Harmonization Guidelines for Good Clinical Practice). Material used in this project was obtained from anonymous rest material. The medical ethics review board waives the need for approval if rest material is used, under the law in the Netherlands and waives the need for informed consent when patient anonymity is assured.

### 4.3. BH3 Profiling—Plate-Based Assay

Cells were incubated at 0.5 × 10^6^ cells/mL for 18 h with AZD1775 (WEE1 inhibitor, Selleckchem No.S1525, Houston, TX, USA) (Figure S8), palbociclib (CDK4/6 inhibitor, Selleckchem No.S1116), RO3306 (CDK1 inhibitor, Selleckchem No.S7747), roscovitine (CDK2 inhibitor, Selleckchem No. S1153), nocodazole (M1404, Sigma Aldrich, St. Louis, MO, USA), or ultraviolet (UV) radiation (IBL 637, CisBioInternational, Gif-sur-Yvette, France). After incubation, cells were washed with mannitol experimental buffer (MEB) (150 mM D-mannitol (M9647, Sigma Aldrich,), 10 mM HEPES (H3375, Sigma Aldrich), 50 mM KCl (1.04936, Merck, Darmstadt, Germany), 20 nM EGTA (E4378, Sigma Aldrich), 20 nM EDTA (11280, Serva Electrophoresis, Heidelberg, Germany), 0.1% BSA (11930, Serva Electrophoresis), 5 mM succinate (1.00682, Merck) in dH2O, pH 7.5) and resuspended at 3.2 × 10^6^ cells/mL in MEB. A cell suspension was mixed 1:1 with 4 µM JC-1 permeabilization/staining solution ((ENZ-52304, Enzo Life Sciences, Farmingdale, NY, USA), 0.004% digitonin (1500 643, Boehringer Mannheim, Mannheim, Germany), 20 mM β-mercaptoethanol (8.05740, Merck) and 40 µg/mL oligomycin (O4876, Sigma Aldrich) prepared in MEB) and incubated at room temperature in the dark for 10 min. BIM, PUMA, BAD, NOXA, MS1, HRK, BMF, and PUMA2A (JPT Peptide Technologies, Berlin, Germany) were prepared in MEB in a black flat-bottom non-treated polystyrene 96-well plate (3915 Costar, Corning Incorporated, Kennebunk, ME, USA). Peptide sequences used for the assay were identical to those described in Ryan and Letai [18]. Plates were either used directly or sealed (Silverseal sealer ref 676090, Greiner-Bio-One, Frickenhausen, Germany), frozen at −80 °C and thawed for 1 h at room temperature before use. Cells in permeabilization/staining solution were added to the plate 1:1 at a final volume of 100 uL and shaken for 15 s, followed by measurement of fluorescence (excitation 545 nm, emission 590 nm) every 5 min for 2 h at 30 °C (Varioskan). All experiments were performed in triplicates or quadruplicate. The area under the curve (AUC) was calculated as a percentage of mitochondrial outer membrane permeabilization (MOMP) and normalized to PUMA2A (negative control) and FCCP (positive control) with the formula: 1 − ((AUC sample − AUC FCCP) ÷ (AUC PUMA2A − AUC FCCP)) × 100%. The dynamic BH3 profile (ΔMOMP) was calculated by subtracting the percentage treated MOMP from percentage untreated MOMP. Based on the specific interacting partners of the BH3 peptides [18], the cell specific anti-apoptotic dependency of cells can be established. BH3 profiling with a mean ΔMOMP ≥ 20% were classified as biologically relevant, even if they were not always statically significant. ΔMOMP changes for the BIM peptide indicates cells have become more primed for apoptosis or closer to their apoptotic threshold. Similarly, ΔMOMP changes for NOXA, HRK, or BAD indicate cells have become more dependent on MCL-1, BCL-XL, or BCL-2/XL/W, respectively. Together, these data can predict on which anti-apoptotic protein cells are dependent, and how this changes upon inhibitor treatment.

### 4.4. BH3 Profiling—Flow Cytometry-Based Assay

Peripheral blood mononuclear cells were isolated from a DLBCL patient, washed with 10% FBS RPMI, incubated at 1.0 × 10^6^ cells/mL for 18 h with 1 µM AZD1775 (WEE1 inhibitor, Selleckchem) in RPMI medium supplemented with 10% fetal bovine serum, 1% Penicillin-Streptomycin, and 1% glutamine. After incubation, cells were washed with 1% BSA (11930, Serva) in PBS and incubated with CD19-APC antibody (IQP-106A, IQ Products, Groningen, The Netherlands) for 30 min at 4 °C. After staining, cells were washed with and suspended in MEB at 3–5 × 10^6^ cells/mL. Cells were incubated with peptides in 0.001% digitonin at 25°C for 60 min. The reaction was stopped by addition of 2% formaldehyde (104005, Merck) in PBS and cells were fixed in 2% formaldehyde for 20 min at room temperature. Cells were washed in 1% BSA in PBS and stained overnight for intracellular cytochrome-c in PBS containing 1% BSA in PBS, 0.2% Tween 20 (P7949 Sigma Aldrich) and 1:400 Alexa Fluor 488 anti-cytochrome-c antibody (612308, BioLegend 9727 Pacific Heights Blvd. San Diego, CA 92121, USA) at 4 °C. Before analysis, cells were washed with 1% BSA in PBS and flow cytometry analysis was performed on a FACSCalibur (BD Biosciences, 1 Becton Drive, Franklin Lakes, NJ 07417-1880, USA). Data were normalized to PUMA2A (negative control) and alamethicin (positive control). The dynamic BH3 profile (ΔMOMP) was calculated by subtracting the percentage treated MOMP from percentage untreated MOMP.

### 4.5. Flow Cytometry-Based Apoptosis Assay

Cells were incubated at 0.1 × 10^6^ cells/mL with AZD1775 (WEE1 inhibitor, Selleckchem) with or without QVD-Oph (pan-caspase inhibitor, Selleckchem, No. S7311) for 18, 24 or 72 h. After incubation, cells were washed with 1% BSA in PBS and stained with Annexin V-FITC (IQP-120F, IQ products, Groningen, The Netherlands) for 20 min on ice. Cells were washed with 1% BSA in PBS and stained with propidium iodide (Sigma Aldrich) to assess early apoptosis (Annexin V positive/propidium iodide negative) and late apoptosis (Annexin V positive/propidium iodide positive). Apoptosis was measured by flow cytometry (FACSCalibur, BD Biosciences). Data were analysed in Winlist 3D (Verity Software house, Topsham, ME, USA).

### 4.6. Flow Cytometry—Cell Cycle, γH2AX, and pH3 with DNA Content

For cell cycle analysis, 0.2 × 10^6^ cells/mL were treated for the indicated time points, washed with 1% BSA/PBS and resuspended in solution containing 0.1% sodium citrate (A0158348, Merck, Kenilworth, New Jersey, USA), 0.01% propidium iodide (P4170, Sigma Aldrich), 0.002% RNase A (R4875, Sigma Aldrich), and 0.3% Triton X100 (T9284, Sigma Aldrich). Samples were processed on a BD FACSCalibur 2 and analysed with ModFit LT (Verity Software House, Topsham, ME , USA).

For γH2AX analysis, 0.2 × 10^6^ cells/ml were treated for the indicated time points and then stained with mouse anti-γH2AX-AlexaFluor-647 (clone 2F3, #613408, BioLegend) and propidium iodide solution (P4170, Sigma Aldrich) according to the protocol provided with the eBioscience™ Foxp3/Transcription Factor Staining Buffer Set (ThermoFisher, #00-5523-00, Waltham, MA, USA). Samples were processed on a MACSQuant and the data were analyzed using Kaluza 1.5 analysis software (Beckman, Brea, CA, USA).

### 4.7. Flow Cytometry-Based Viability Assay

Cells were incubated at 0.1 × 10^6^ cells/mL with AZD1775 (WEE1 inhibitor, Selleckchem) for 18 h. After washing with 1% BSA in PBS, cells were incubated with venetoclax/ABT-199 (BCL-2-selective inhibitor, Selleckchem), S63845 (MCL-1 inhibitor, Selleckchem), or navitoclax/ABT-263 (BCL-XL/BCL-2/BCL-W inhibitor Selleckchem) for 48 h at 37 °C. After incubation, cells were washed with 1% BSA in PBS and stained with propidium iodide (Sigma Aldrich) to assess cell viability by flow cytometry (FACSCalibur, BD Biosciences). Data were analysed in Winlist 3D (Verity Software House, Topsham ME, USA).

### 4.8. Statistical Analysis

A one-sample *t*-test was used to assess the significance of the dynamic BH3 profiling results (*n* = 3 independent repeats). Comparison of IC50 values between untreated and AZD1775-treated samples was done using a two-tailed paired *t*-test. Correlation analysis was carried out using Pearson’s correlation test. * indicates *p* ≤ 0.05, ** indicates *p* ≤ 0.01, and *** indicates *p* ≤ 0.001. 

## 5. Conclusions

In conclusion, we demonstrate that treatment of cells with WEE1 inhibitor AZD1775 can enhance the dependency on anti-apoptotic proteins and enhanced sensitivity to BH3 mimetic drugs. In addition, we showed that DNA damage and cell cycle arrest independently induce similar changes in the anti-apoptotic dependency. Therefore, we predict that other DNA damage-inducing of cell cycle arresting agents will synergize with BH3 mimetic drugs.

## Supplementary Materials

The following are available online at https://www.mdpi.com/2072-6694/11/11/1743/s1. Table S1: Characteristics of DLBCL cell lines, Table S2: BH3 treatment schedule for DLBCL cell lines, Figure S1: Gene expression levels of WEE1 in multiple cancer types, Figure S2: Time and dose-dependent induction of apoptosis by AZD1775 in DLBCL, Figure S3: Representative example of the dose-response effect of AZD1775 on mitochondrial outer membrane permeabilization, Figure S4: DNA damage induced by AZD1775, nocodazole, or UV treatment, Figure S5: DNA damage induced by AZD1775 alone or combined with CDK inhibitors, Figure S6: Correlation of dynamic BAD BH3 profile and γH2AX after Palbociclib and AZD1775, Figure S7: DLBCL patient characteristics. Figure S8: DLBCL cells treated with AZD1775.
