# Supplementary material for: WEE1 Inhibition Enhances Anti-Apoptotic Dependency as a Result of Premature Mitotic Entry and DNA Damage
Source: Cancers (Basel). 2019 Nov 7;11(11):1743. doi: 10.3390/cancers11111743 (PMC6895818; doi:10.3390/cancers11111743)
Supplement: Supplementary file 1 [file cancers-11-01743-s001.pdf]

Supplementary Materials

WEE1 Inhibition Enhances Anti-Apoptotic Dependency as a Result of Premature Mitotic Entry and DNA Damage

Mathilde Rikje Willemijn de Jong [1,2], Myra Langendonk [1,2], Bart Reitsma [1], Pien Herbers [1], Marcel Nijland [1], Gerwin Huls [1], Anke van den Berg [2], Emanuele Ammatuna [1], Lydia Visser [2] and Tom van Meerten [1,*]

Table S1. Characteristics of DLBCL cell lines. Characteristics of the DLBCL cell lines and their cell of origin (COO), IC50 value for AZD1775, TP53 mutation status and cytogenetic profile (DSMZ/ATCC).

Cell line	COO subtype	AZD1775 IC50 (µM)	TP53 status	TP53 mutation	Cytogenetics (DSZM or ATCC)
OCI-LY3	ABC	0.68	WT	x	Human flat-moded hypertriploid karyotype; 72-77 < 3n > XXYY, +1, +9, -10, +13, +14, -17, +19, +20, +22, der(1)t(1;17)(p13;q12)x2, der(4)t(4;18)(q31;q21)x2, del(6)(q13)x2, der(6)t(6;6)(p24;q12), der(7)t(6;7)(p24;p22), der(14)t(14;19)(q32;q13.3)x2, del(18)(q21), der(19)t(4;19)(q21;q13)t(4;18)(q31;q21)x2, der(19)t(14;19), dup(20)(q11q13)x2; sdl with der(6)t(6;12)(p21;q21), der(7)t(5;7)(?p15;p24) etc; resembles published karyotypes; carries cryptic t(14;19) with rearrangement of IGH and SPIB, and t(4;18) with copy number amplification of the BCL2 region.
U-2932	ABC	1.02	MUT	c.527G > A	Human polyclonal hypodiploid karyotype with 20% polyploidy; 45(43-46) < 2n>, XX, add(X)(q22), add(1)(q24), der(3)ins(3;18)(q27;q2?q2?)hsr(18)(q21), add(5)(q32), der(6)t(6;18)(p24;?q23)del(6)(q13), der(10)t(10;14)(q24;q23), der(11)t(1;11)(q25;q2?), der(14)t(3;14)(q27;p11), der(18)t(1;18)(q21;q21), der(18)t(3;18)(q2?;q27), add(19)(p13), del(19)(q13); sdl with del(2)(q11), t(4;15)(q22;q14), add(7)(q21), del(13)(?q21), der(13)add(13)(p11)add(13)(q32); extensive genomic amplification of BCL2 region; resembles published karyotype.
SUDHL-2	ABC	0.42	MUT	c.701A > C	Most of these cells were hyperdiploid, with a sharp modal number of 51 chromosomes; occasional cells in the hypertetraploid range were also seen. A minute marker

					chromosome was consistently observed, and increased numbers of chromosomes were seen in the A, B, C, and F groups.
SUDHL-4	GCB	0.99	MUT	c.817C > T	Human hyperdiploid karyotype with 4% polyploidy - 50(47-51) < 2n > XY/XXY, -3, +7, +8, +12, +13, -16, +18, der(3)t(3;3)(p2?4;p1?2), der(8)t(3;8)(p1?2;q12)?inv(3)(p1?p2?), der(12)t(3;12)(p1?;q24)add(3)(p2?4), add(13)(p11), t(14;18)(q32.3;q21.1), add(16)(p13), der(18)t(14;18)(q32.3;q21.1) - carries t(14;18) effecting BCL2(MBR)-IGH fusion - matches published karyotype.
SUDHL-5	GCB	0.81	WT	x	Human hyperdiploid karyotype with 1.5% polyploidy - 47(41-48) < 2n > XX, +12, del(6)(q13), del(12)(q13) - sideline with del(6)x2 - matches published karyotype.
SUDHL-6	GCB	1.42	MUT	c.701A > G	Human hyperdiploid karyotype with 2% polyploidy - 47(42-48) < 2n > X, -Y, +6, +7, del(4)(q23), del(6)(p21.3p22.2), i(6p), del(7)(q?22q?32), der(8)t(8;9)(q24;p13), der(9)t(8;19;9)(q24;q13;p13), dup(11)(q24q25), t(14;18)(q32;q21), der(22)t(?7;22)(?q32;p11) - sideline with dic(8;9)(q24;p13), ider(8)(q10)t(8;9(q24;p13) - matches published karyotype - carries t(14;18) effecting IGH-BCL2 fusion.
SUDHL-10	GCB	0.79	MUT	c.994-1G > C	Human flat-moded hyperdiploid karyotype - 47(43-48) < 2n > XY, +7, der(8)t(X;8)(q25;p23)t(8;X)(q24;q26)t(X;14)(q28;q32), del(10)(q22q24), der(11)t(Y;11)(q11;q25), der(14)t(8;14)(q24;q32), der(18)t(14;18)(q32;q21) - carries concurrent rearrangements of IGH with MYC and BCL2 - resembles published karyotype.
SC-1	GCB	0.83	WT	x	Human hyperdiploid karyotype with 4% polyploidy - 47(43-48) < 2n > XY, +7, -17, +mar, add(3)(q27), t(8;14;18)(q24;q32;q21), add(14)(q32) - carries 3-way t(8,14,18) effecting genomic co-amplification of BCL2 and MYC - resembles published karyotype.

Table S2. BH3 treatment schedule for DLBCL cell lines. Schematic overview of the treatment schedule applied to the DLBCL cell lines in order to establish the effect of AZD1775 on anti-apoptotic dependency via dynamic BH3 profiling.

Cell line	AZD1775 (μM)	BIM (μM)	NOXA (μM)	HRK (μM)	BAD (μM)
OCI-LY3	0.5	0.1	10	10	0.1
U-2932	0.5	0.1	10	10	0.1
SUDHL-2	0.5	0.1	10	10	10
SUDHL-4	1	0.1	10	10	0.1
SUDHL-5	0.5	0,1	10	10	10
SUDHL-6	1	0.03	10	10	1
SUDHL-10	0.25	0.1	10	10	10
SC-1	1	0.3	10	10	0.1

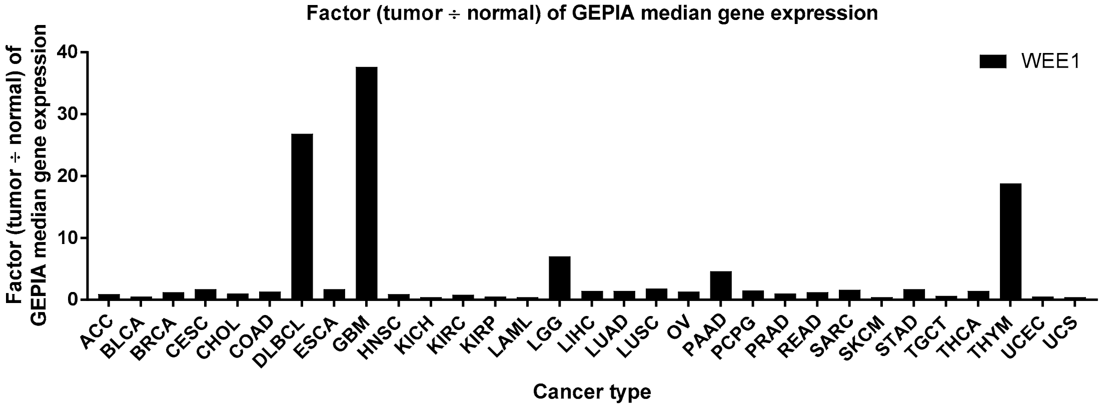

Figure S1. Gene expression levels of WEE1 in multiple cancer types. Plotted is the factor of tumor tissue / representative normal tissue gene expression for WEE1 across multiple types of cancers. Cancers included are adrenocortical carcinoma (ACC), bladder urothelial carcinoma (BLCA), breast invasive carcinoma (BRCA), cervical squamous cell carcinoma and endocervical adenocarcinoma (CESC), cholangio carcinoma (CHOL), colon adenocarcinoma (COAD), diffuse large B-cell lymphoma (DLBCL), esophageal carcinoma (ESCA), glioblastoma multiforme (GBM), head and neck squamous cell carcinoma (HNSC), kidney chromophobe (KICH), kidney renal clear cell carcinoma (KIRC), kidney renal papillary cell carcinoma (KIRP), acute myeloid leukemia (AML), brain lower grade Glioma (LGG), liver hepatocellular carcinoma (LIHC), lung adenocarcinoma (LUAD), lung squamous cell carcinoma (LUSC), mesothelioma (MESO), ovarian serous cystadenocarcinoma (OV), pancreatic adenocarcinoma (PAAD), pheochromocytoma and paraganglioma (PCPG), prostate adenocarcinoma (PRAD), rectum adenocarcinoma (READ), sarcoma (SARC), skin cutaneous melanoma (SKCM), stomach adenocarcinoma (STAD), testicular germ cell tumors (TGCT), thyroid carcinoma (THCA), Thymoma (THYM), uterine corpus endometrial Carcinoma (UCEC), uterine carcinosarcoma (UCS) and uveal melanoma (UVM). Data was acquired from the online available GEPIA database (gepia.cancer-pku.cn).

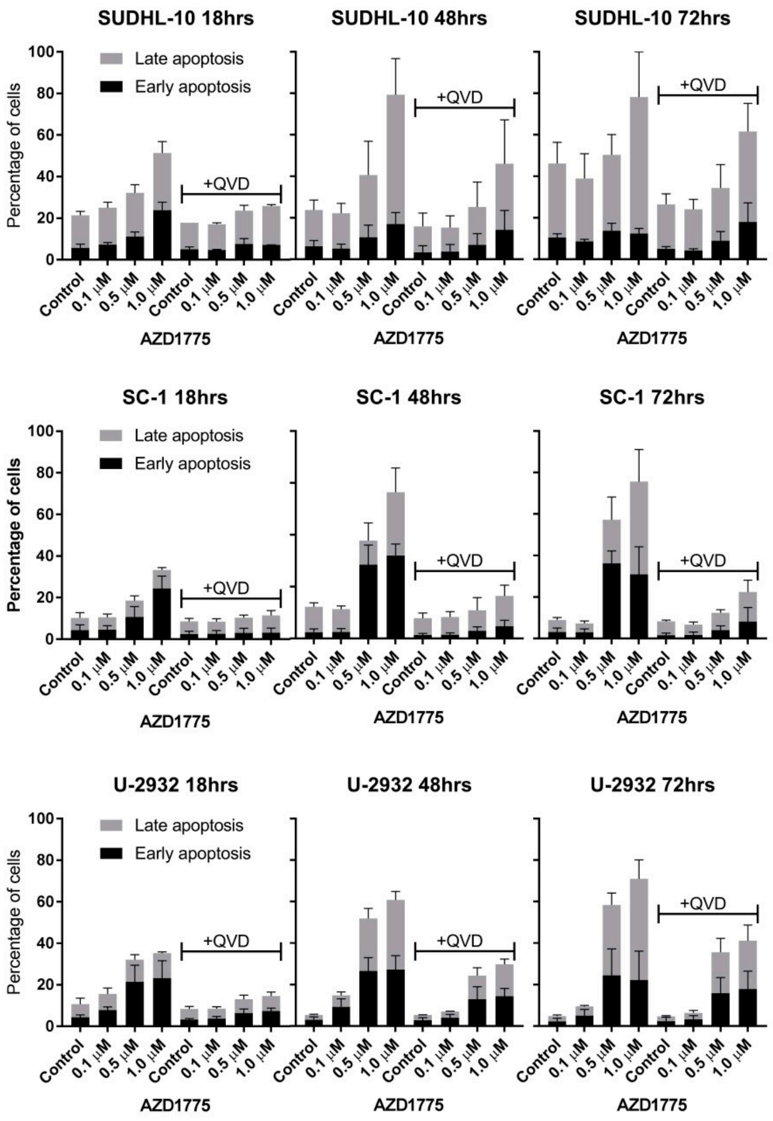

Figure S2. Time and dose-dependent induction of apoptosis by AZD1775 in DLBCL. Flow cytometry analysis of early apoptosis (Annexin V positive/Propidium iodide negative) and late apoptosis (Annexin V positive/Propidium iodide positive) in cell lines SUDHL-10, SC-1 and U-2932 treated with AZD1775 ± 20 μM caspase inhibitor QVD for 18, 24 or 48 h. Plotted is the mean of *N* = 3.

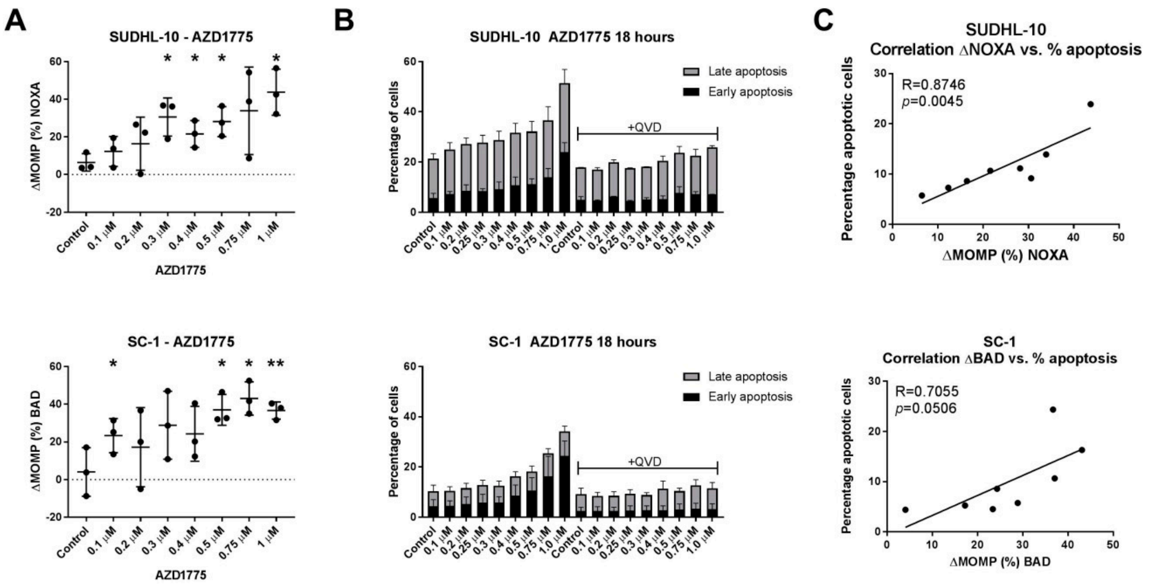

Figure S3. Dose-response effect of AZD1775 on mitochondrial outer membrane permeabilization. (**A**) Dynamic BH3 profile of cell lines SUDHL-10 and SC-1 cell line treated with an increasing concentration of AZD1775 for 18 h. Delta mitochondrial outer membrane permeabilization (ΔMOMP%) for 10 μM NOXA for cell line SUDHL-10 and 0.1 μM BAD for cell line SC-1 was calculated by subtracting the percentage treated MOMP from percentage untreated MOMP. Data were plotted as the mean ± SD ($N = 3$). Statistical analysis was performed using a one-sample T-test as compared to untreated control cells (*$p \leq 0.05$). (**B**) Flow cytometry analysis of early apoptosis (Annexin V positive/Propidium iodide negative) and late apoptosis (Annexin V positive/Propidium iodide positive) in cell lines SUDHL-10 and SC-1 treated with AZD1775 ± 20 μM caspase inhibitor QVD for 18 h. Plotted is the mean of $N = 3$. (**C**) Correlation of the ΔMOMP% for 10 μM NOXA in SUDHL-10 and 0.1 μM BAD in cell line SC-1 and the percentage of early apoptotic cells (Annexin V positive/Propidium iodide negative) after 18 h of AZD1775 treatment.

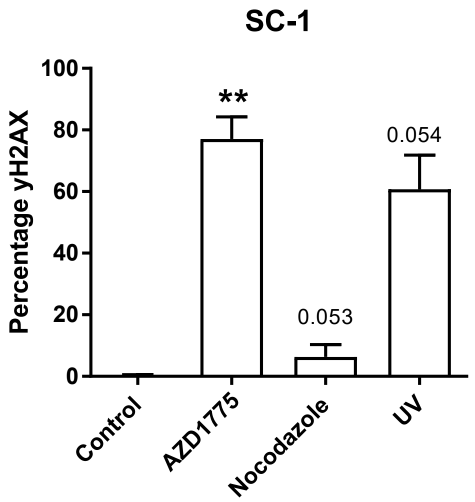

Figure S4. DNA damage induced by AZD1775, nocodazole or UV treatment. Percentage of yH2AX positive cells after treatment of cell line SC-1 with 1 μM AZD1775, 50ng/mL nocodazole or 20 J/m^2 after 18 h of incubation. Data were plotted as the mean ± SD ($N = 3$). Statistical analysis was performed using a two-tailed paired T-test comparing treatment to control (*$p \leq 0.05$) (**$p \leq 0.01$).

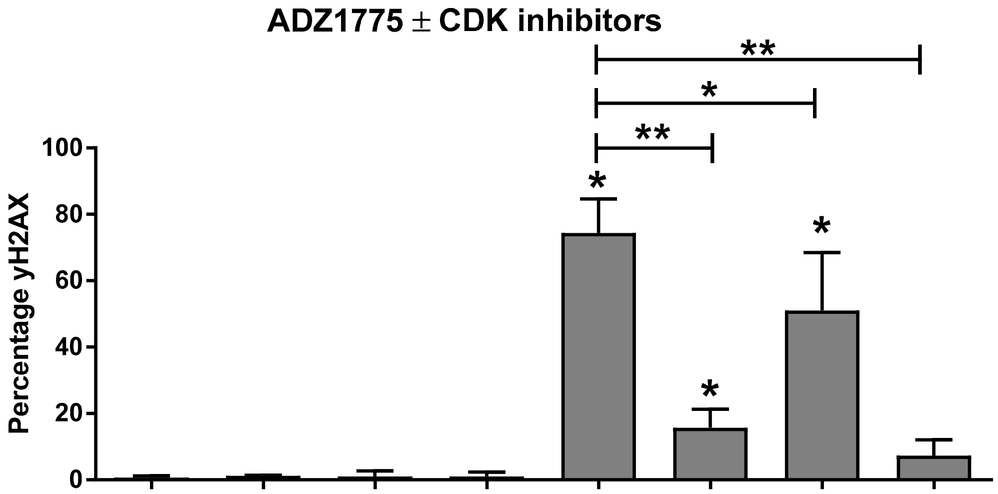

AZD1775	-	-	-	-	1µM	1µM	1µM	1µM
RO3306	-	10µM	-	-	-	10µM	-	-
Palbociclib	-	-	1µM	-	-	-	1µM	-
Roscovitine	-	-	-	15µM	-	-	-	15µM

Figure S5. DNA damage induced by AZD1775 alone or combined with CDK inhibitors. Percentage of yH2AX positive cells after treatment of cell line SC-1 with 10 µM RO3306, 1 µM palbociclib, 15 µM roscovitine and 1 µM AZD1775 after 18 h of incubation. Data were plotted as the mean ± SD (*N* = 3). Statistical analysis was performed using a two-tailed paired *T*-test comparing treatment to control (*$p \leq 0.05$) (**$p \leq 0.01$).

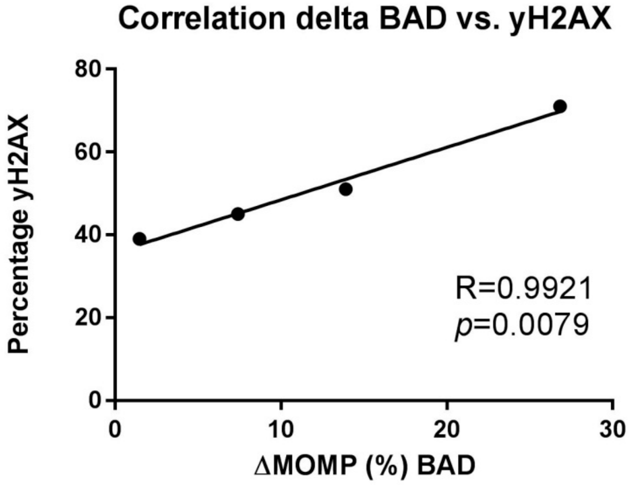

Figure S6. Correlation of dynamic BAD BH3 profile and γH2AX after Palbociclib and AZD1775. (**A**) Correlation of the (ΔMOMP%) for 0.1 µM BAD versus the percentage of γH2AX cells in cell line SC-1 cells treated with AZD1775 and palbociclib for 18 h.

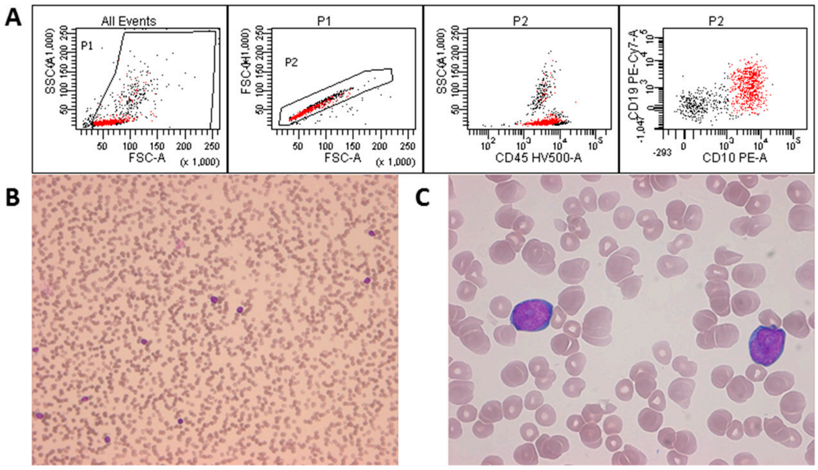

Figure S7. DLBCL patient characteristics. (**A**) Flow cytometry analysis of bone marrow aspirate. (**B**) Smear of bone marrow aspirate and (**C**) enlarged image of bone marrow aspirate smear. Depicted DLBCL patient sample contains 60% CD10+/CD19+/CD45+ monoclonal lymphoid B-cells.

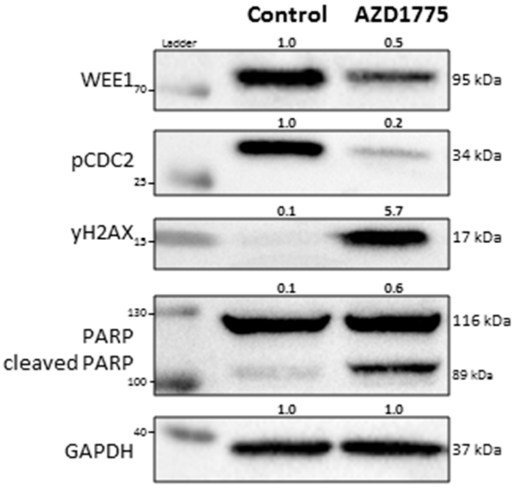

Figure S8. DLBCL cells treated with AZD1775. Representative DLBCL cell line U-2932 was treated with 0.5 µM AZD1775 for 24 h. Protein analysis was performed for WEE1, phospho-CDC2 (Tyr15) for WEE1 activity, yH2AX and cleaved PARP.

© 2019 by the authors. Licensee MDPI, Basel, Switzerland. This article is an open access article distributed under the terms and conditions of the Creative Commons Attribution (CC BY) license (http://creativecommons.org/licenses/by/4.0/).
